# On the Requirements for a Reasonable Extreme Value Prediction of Maximum Pits on Hot-Water-Supply Copper Tubing

**DOI:** 10.6028/jres.099.029

**Published:** 1994

**Authors:** Shigeru Komukai, Komei Kasahara

**Affiliations:** Tokyo Gas Company, Ltd., Fundamental Technology Research Laboratory, 1-16-25 Shibaura, Minato-ku, Tokyo 105, Japan

**Keywords:** central hot-water-supply, copper tube, extreme value statistical analysis, maximum pit depth prediction, pitting corrosion

## Abstract

Application of extreme value statistics to the problem of Type-II pits growth prediction on hot-water-supply copper tubing is described. A recommendation is suggested for optimum combinations of the number and the size of unit samples required for reasonable extreme value predictions.

## 1. Introduction

Modern large buildings in the Tokyo metropolitan area usually have a centralized hot-water supply operating 24 h a day. Copper pipe is widely used in such systems because of its relatively high resistance to corrosion, coupled with additional pragmatic merits: it is easy to work with, easy to install, and relatively cheap.

The seriousness of Type-II pitting corrosion, however, has increasingly received high recognition in such hot-water supply systems [1,2,3]. The need to obtain information regarding the degree of pitting corrosion has increased over the last decade because considerable pipe damage may require maintenance, and even replacement, and in that case, proper life prediction is essential to pass reasonable engineering judgements and thereby to perform proper maintenance.

The life prediction of such copper plumbing tubing can first be performed by coupling adequate nondestructive and/or, though less favorable, destructive inspection techniques with reliable statistical analysis. It has been shown that the most promising statistical analysis methods for such a purpose include extreme value statistical analysis [3,4].

Although such tools have become widespread, a general method for evaluating the localized corrosion propensity on existing engineering structures from limited inspection data, and concrete criterion for the number and size of samples required to obtain a reasonable extreme value prediction is still not available.

In the present study, a set of pitting corrosion depth data obtained from 7 year old copper plumbing pipe, one third of which was removed from a centralized hot-water supply system, was examined by extreme value statistics. Emphasis was placed on the effect of the total number and size of maximum pit depths on the accuracy of pit growth prediction. A concept was ultimately proposed to obtain a reasonable prediction of maximum pit depths while minimizing the total sampling area (or, length) for analysis.

## 2. Test Procedures

### 2.1 Test Specimens

Copper pipes totaling 8.88 m in length were removed from various parts of the centralized hot-water supply system in an 11-story multi-family dwelling in Tokyo. The system consisted of one stainless steel storage tank (1.8 m^3^) and copper plumbing pipe having an overall length of about 28 m. The plumbing material was JIS C 1220T type 25AM (outside diameter 28.58 mm and wall thickness 0.89 mm) copper pipe for building use. The system had been operating for about 2600 d before test piping was removed. In the system, water at a nominal temperature of 60°C circulated constantly. Average flow (i.e., hot-water consumption) was around 8 m^3^/d, the storage tank being supplied automatically with tap water.

The copper piping removed was cut into parts 100 mm long, which were then cut in half to give half-ring specimens. Each half-ring specimen was then completely cleaned ultrasonically in dilute sulfuric acid, followed by marking-off to divide it into 10 virtual half-ring specimens of 10 mm unit length, after which the pit depths were measured by using an optical microscope of 1 μm precision. By coupling two opposite virtual half-ring specimens, a 10 mm long full ring specimen was reassembled, and the area thus surveyed should be representative of the pipe at that particular location. Then, by taking several adjoining full-rings of 10 mm length, for each unit sample sizes of maximum pit depths (hereinafter, s: unit length) in the interval 20 mm up to 200 mm were obtained.

### 2.2 Extreme Value Statistical Analysis

The extreme value statistical analysis was performed by using a commercial available personal computer software package, EVANS [5].

The basic concept of the present extreme value analysis is briefly reviewed in the following sections.

#### 2.2.1 Extreme Value Probability Plots

The first step of the extreme value analysis included the preparation of extreme value probability plots, that is, plots of maximum pit depth data on extreme value probability paper of the cumulative relative frequency (*F*(*y*)) vs maximum pit depth (*x*). Maximum pit depths data were arranged in order from largest to smallest and assigned a rank number. The vertical plotting position *F*(*y*) for each pit depth value was calculated by the averaged rank method as follows:

F(y)=1−i/(N+1) (1)

where

*i* = rank number,
*N* = total number.

Several sets of plots were obtained depending on the combinations of *N* and *s*. In an exemplifying case of *s* = 100 mm and *N* = 7, a total of 10 sets of plots was subjected to regression analysis.

#### 2.2.2 Regression for the Best Fit Line

Regression analysis based on the MVLUE (Minimum Variance Linear Unbiased Estimator) method was made for each data set to determine a straight line of best fit to the plotted extreme values. The equation of a straight line is given as follows:

x=λ+αy (2)

where

**Fig. 1:**
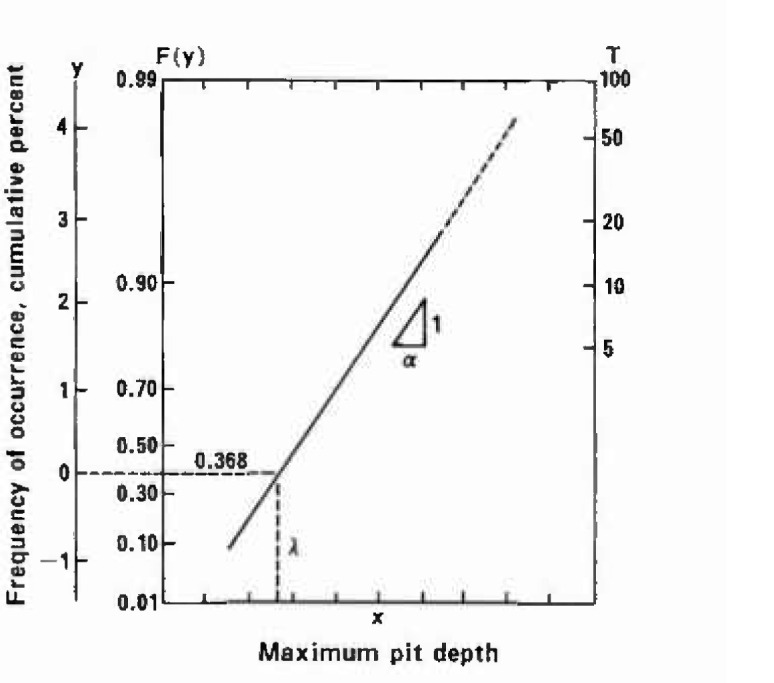
Extreme value probability paper of double-exponential distribution.

*x* = expected maximum pit depth,
*y* = standardized variable,
λ = location parameter,
*α* = scate parameter,

The straight line is drawn in Fig. 1 on extreme value probability paper. In the present analysis, emphasis was placed on the optimum combinations of *N* and *s* for obtaining a reasonable estimate of the extreme value. Such an analysis becomes feasible through a thorough investigation of all the samples where the depth of the actually detected deepest pit could be regarded as the probable maximum pit depth, that is, the extreme value.

## 3. Results and Discussion

### 3.1 Distribution of Pit Depths

**Fig. 2:**
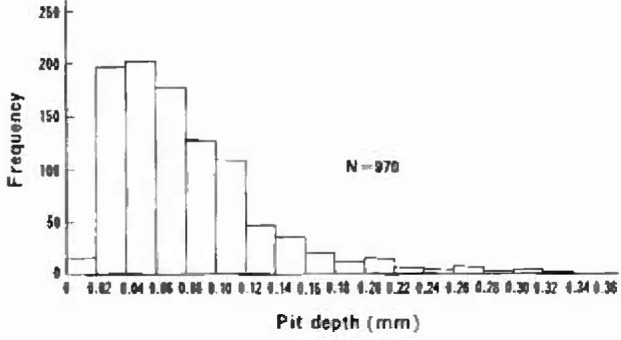
Pit depths distribution histogram for 10 mm long copper pipe used for 2600 d in a hoi-water supply system operated at 60°C.

Figure 2 is a histogram showing the relationship between the pit depths and the total frequency. In the figure, a total of 970 depth data for all pits found in one 10 mm long full-ring, removed from -the 7th floor in the building, was grouped over the pit depth ranges, 0 mm–0.019 mm, 0.020 mm–0.039 mm, etc. As is evident from the Figure, the shape of the pit depth distribution is a bell-shaped curve starting at zero, rising to a maximum at around 0.05 mm and thereafter decreasing rapidly with increasing pit depths. The lack of “J”-shaped portion bending to the right in the pit depth range from 0 mm to 0.02 mm, together with the trailing extreme portion of the tail of the curve up to 0.34 mm, indicated that most of the small pits had already had ceased to grow while only a small number of deeper pits continued to grow.

**Fig. 3:**
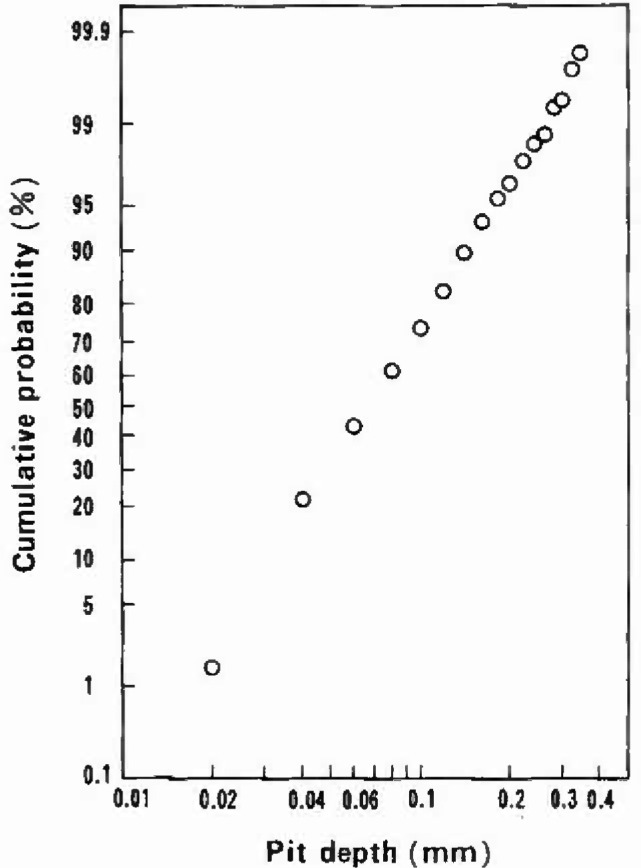
Plot on a logarithmic-normal distribution diagram of all data presented in Fig. 2.

In Fig. 3, all the pit depth data represented in Fig. 2 were plotted on a logarithmic-normal distribution diagram. The apparent linearity of the plot indicates that this distribution applies to the logarithmic-normal; and hence the maximum pit depth data obtained in the present pit depth survey were sampled from the parent populations with a logarithmic-normal distribution.

**Fig. 4:**
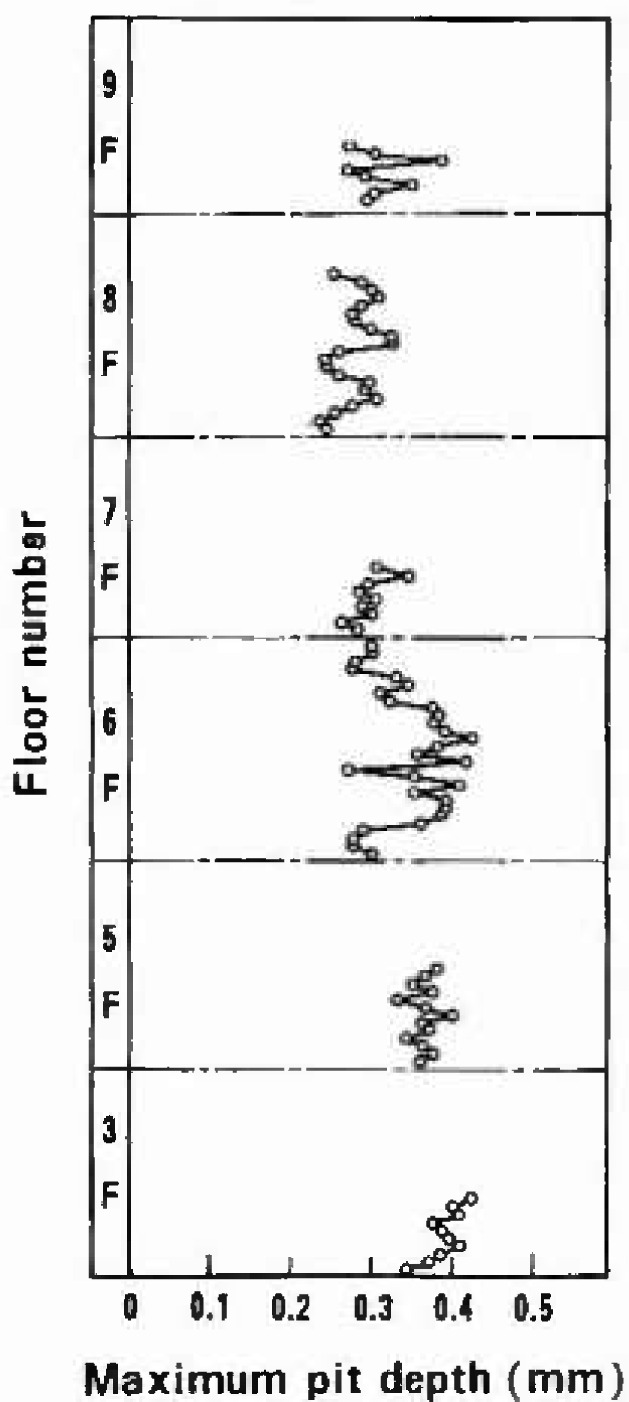
Distribution at each floor in the building of the maximum pit depth detected in each 100 mm unit length.

Figure 4 shows the distribution at every floor of the maximum pit depth detected in each 100 mm unit length. Though the maximum pit depths seemed to have a slight tendency to become shallower at upper floors, the distribution of pit depths was regarded as being uniform throughout the building. The actual maximum pit depth value in the present survey was 0.452 mm which was detected at 6th, floor.

**Fig. 5:**
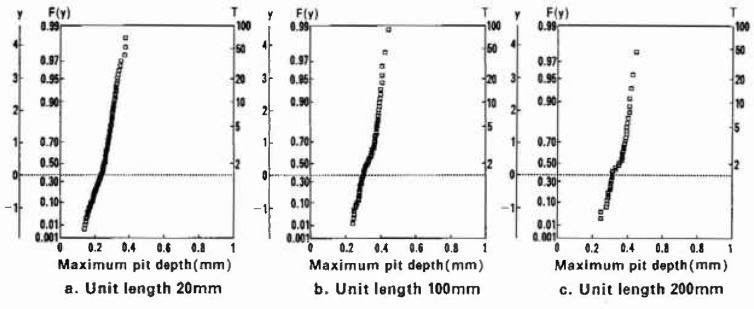
Double-exponential distribution type of extreme value probability plots for *s* = 20 mm, 100 mm, and 200 mm.

In Figure 5, the maximum pit depth data for unit lenghs of 20 mm, 100 mm, and 200 mm were evaluated from extreme value analysis on the basis of Gumbel’s double-exponential distribution; the linearity of each plot shows that this distribution applies to the maximum pit depth data obtained at the unit lengths between 20 mm and 200 mm.

### 3.2 Minimum Required *N* and *s*

In the practical application of extreme value statistics, the number and the size of unit samples for the pit depths survey are to be decided prior to the destructive (or nondestructive) inspection of the existing structures.

Reliability of the extreme value prediction depends on the following three equations;

y=lnT (3)

T=S/s (4)

$$V\left(x\right)=\alpha^{2}\left[A\left(N,n\right)y^{2}+B\left(N,n\right)y+C\left(N,n\right)\right]$$ (5)

where

*y* = standardized variable,
*T* = return period,
*S* = total length,
*s* = unit length,
*V*(*x*) = variance of the estimated extreme value,
*A*(*N, n*), *B*(*N, n*), *C*(*N*, *n*) = MVLUE coefficients,
*α* = scale parameter.

It is obvious from these equations that for increased reliability, hence for a minimized *V*(*x*), it is required to increase *s* (therefore to decrease *T*) and/or to increase *N*. In the interest of economy, however, there must be a natural limitation to the increase of *N* and *s* for the extreme value survey.

In theory, the optimum combinations of *N* and *s* can be determined from Eqs. (3) to (5) once the distribution parameter (that is, a ratio of the location parameter to the scale parameter, *α*/λ) is reasonably assumed (that is, empirically or experimentally), and the extent of the standard deviation of the error of extreme value estimates, *σ*, may be expected to be:

λ=mσ (6)

where

λ = location parameter,
*σ* = standard deviation of the estimated extreme value,
*m* = assumed number (1, 2, 3, etc.)

That is, the decision may be made to reduce *σ* to 1/m of λ [6].

Table 1 shows the result of extreme value analysis to obtain the relationship between unit length and distribution parameter. In the analysis, all pit depth data obtained from the whole lengths were brought into consideration. It can be seen in the table that the distribution parameter, *α*/λ, decreased with increasing unit length, ultimately approaching to a definite level of 0.15.

**Fig. 6:**
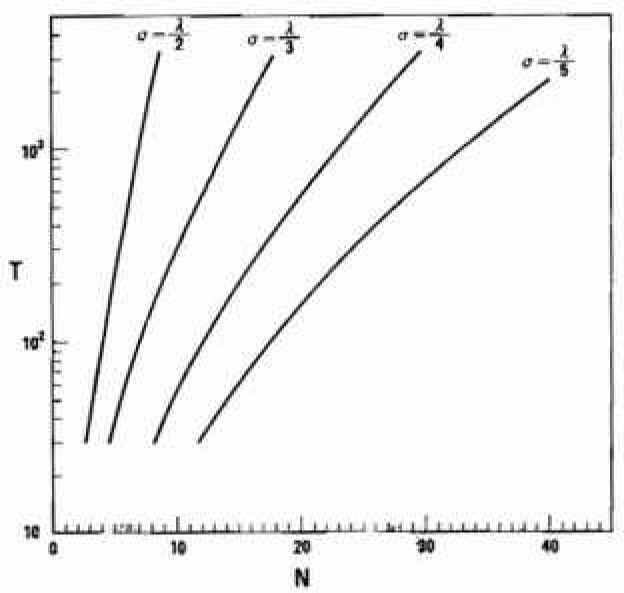
Optimum combinations of *N* and *T* to control *σ* at levels of 1/2, 1/3, 1/4, or 1/5 of λ under the limitation of *α*/λ =0.2.

Optimum combinations of *N* and *T* (hence, *s*) required for controlling *σ* down to 1/*m* of λ were obtained as shown in Fig. 6 corresponding to *α*/λ of 0.15 and 0.20. It stands to reason, that the number of unit lengths, *N*, can markedly be reduced by loosening the requirement for the reliability, that is, by decreasing *m. N* may also be reduced by decreasing *α*/λ. Though *N* may be reduced by increasing *s* (hence decreasing *T*), it should be noted that the total length required for a pit depths survey can increase on the contrary.

### 3.3 Maximum Pit Depths Prediction Based on the Optimized *N* and *s*

**Fig. 7:**
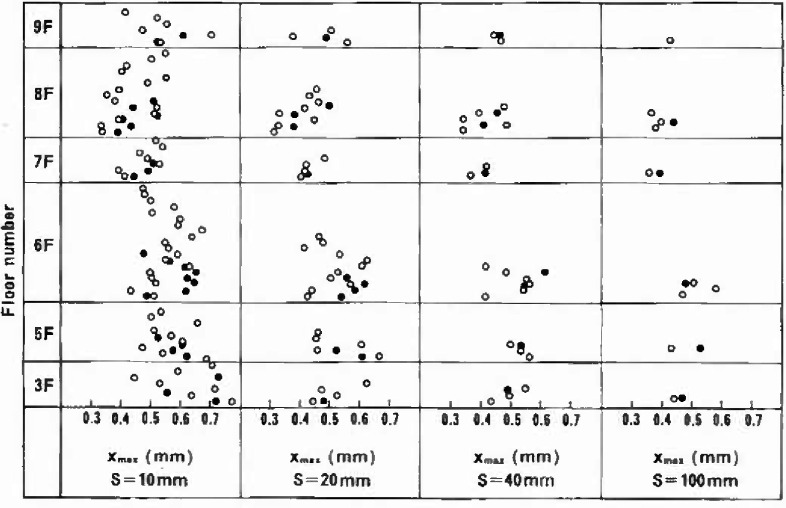
Distribution at each floor of the estimates of maximum pit depths on the basis of the optimum combinations of *N* and *T* under the limitations of λ =3*σ* and *5σ*.

Figure 7 shows the results of the maximum pit depths prediction by using the combinations of *N* and *s* from Fig. 6. Unit lengths for this evaluation were not sampled at random locations, but were ordered from one end of the lower floors upward. Thus the results were represented by correlating to the locations wherefrom those unit lengths used in the prediction were removed. It can be seen that the scatter of estimated extreme values decreased with increasing *s*. As would be expected, the scatter was narrower at *m* = 5 as compared with *m* = 3.

To determine the general tendency in the reliability of the present extreme value prediction, Table 2 was developed from data on Fig. 7 by taking an average for each item. The results indicate the following:

1. The assumptions for *α*/λ and *m*, that were made prior to the analysis, have conservatively been met.
2. Increased *m* did not always result in increased reliability which indicated that *m* = 3 might be reasonable in the interest of economy.
3. Except for the cases of *s* = 10 mm, the maximum detected pit depth value of 0.452 mm fell closely between the average of estimates *±* 1*σ*.
4. Although the level of *s* is to be kept as low as possible, because the total sampling length (ie., *N* × s) may increase with increasing *s*, at least 2.5% of the whole structure is to be subjected to extreme value evaluation at a return period of 500 or the less.

## 4, Concluding Remarks

Based on this analysis, the following conclusions can be drawn concerning the optimum conditions for obtaining a reasonable extreme value prediction.

1. The number and the size of unit samples for the extreme values survey may be determined so that the variance of the extreme value estimates is to be minimized under a definite distribution parameter, wherein the standard deviation of the estimates is expected to be 1/3 of the mode of distribution.
2. A return period of 500 with a total sampling length amounting to 2.5% of the entire parts may be desirable for an increased reliability of extreme value prediction.

## Figures and Tables

**Table 1:** Location, scale, and distribution parameters determined from all maximum pit depth data obtained in the survey

Unit length (mm)	*T*	*N*	*x*_max_ (mm)	λ (mm)	*α* (mm)	*α*/λ
50	177.6	172	0.575	0.28415	0.05621	0.198
100	88.8	36	0.528	030446	0.04986	0.16
200	44.4	43	0.506	0.3221	0.04866	0.151

**Table 2:** Summary of the extreme value prediction

	Unit length *S* (mm)	Return period T	Sample number *N*	Location parameter λ_av_	Scale parameter *α*_av_	Estimated maximum pit depth ${\hat{X}}_{\max}$	$\bar{\sigma }x_{\max}$	$\frac{\lambda_{xv}}{\bar{\sigma }x_{\max}}$
λ = 3*σ*	10	888	14	0.241	0.043	0.534	0.069	3.50
	20	444	12	0.269	0.034	0.478	0.054	4.98
	40	222	9	0.288	0.035	0.467	0.058	4.98
	100	88.8	7	0.313	0.028	0.428	0.045	6.94

λ = 5*σ*	10	888	33	0.218	0.046	0.559	0.047	4.63
	20	444	28	0.266	0.040	0.491	0.040	6.67
	40	222	23	0.282	0.039	0.487	0.039	7.25
	100	88.8	18	0.312	0.034	0.463	0.032	9.71
